# A novel peptide ‘T14’ reflects age and photo-aging in human skin

**DOI:** 10.18632/aging.204844

**Published:** 2023-06-28

**Authors:** Sheila Rocha, Sara Garcia Ratés, Tumisang Moswete, Kristopher Kalleberg, Anna Villa, Jason P. Harcup, Susan A. Greenfield

**Affiliations:** 1 Unilever Research and Development, Trumbull, CT 06611, USA; 2 Neuro-Bio Ltd, Culham Science Center, Abingdon, Oxfordshire OX14 3DB, UK

**Keywords:** skin, age, photo-aging, acetylcholinesterase peptide, keratinocyte

## Abstract

T14 is a 14mer peptide derived from the C-terminus of acetylcholinesterase (AChE). Once cleaved, it is independently bioactive of the parent molecule and enhances calcium influx in different cell types, in a range of scenarios: it binds to an allosteric site selectively on the alpha-7 receptor, where it modulates calcium influx and is thus a potential trophic agent, as already reported in a range of normal developmental scenarios. However, if inappropriately activated, this erstwhile beneficial effect converts to a toxic one, resulting in pathologies as disparate as Alzheimer’s and various metastatic cancers. Given that epidermal keratinocyte cells have the same ectodermal origin as brain cells, as well as expressing AChE and the alpha-7 receptor, we have explored whether T14 plays a comparable role. Here we report that the T14 immunoreactivity is detectable in human keratinocytes with levels inversely related to age: this decrease is even more apparent with chronic photo-exposure and thus accelerated skin aging. We conclude that T14, an agent promoting cell growth and renewal in other parts of the body, also operates in skin, Moreover, monitoring of keratinocyte T14 levels might offer further insights into the now well reported link between degenerative diseases and epidermal cell profile.

## INTRODUCTION

Skin aging is a multifactorial process driven by genetic, environmental and lifestyle factors affecting a variety of molecular mechanisms. High-turnover tissues, like the epidermal layer of the skin, are dependent on the ability to self-review to preserve proper function and thus aging-induced changes in pathways regulating these mechanisms can lead to aging-associated pathologies [1]. A dominant feature of the epidermis and dermis is the alpha 7 nicotinic acetylcholine receptor (α7nAChR): this powerful calcium ionophore plays a critical role in skin biology, skin aging and photo-aging, as it impacts on keratinocyte differentiation and directional migration, inflammatory and oxidative stress, as well dermal remodeling [2, 3]. In addition, an α7nAChR agonist has been shown to suppress UV induction of proinflammatory cytokines and oxidative stress, mechanisms that play a key role in skin photo-ageing. In the epidermis, the α7nAChR is strongly expressed in upper stratum spinosum and granulosum [2, 3] where it mediates regulation of keratinocyte biology via acetylcholine signaling downstream [3]. As might be expected of classic cholinergic transmission, the expression of the α7nAChR overlaps with that of the enzyme that hydrolyses acetylcholine, acetylcholinesterase (AChE), found in epidermal keratinocytes in the basal, spinous and granular cell layers [4, 5] as well as in dermal fibroblasts [6].

However, as an alternative primary ligand to acetylcholine, dietary derived choline can instead complex with the α7nAChR [7, 8] and could mediate a second signaling system in parallel to, and independent of, classic cholinergic transmission [9]. In support of this general notion exemplified in the specific context of the skin, keratinocytes express two forms of AChE, a tetrameric membrane anchored protein ‘G4’ that would adequately meet the requirements for classic cholinergic transmission and the AChE monomeric form ‘G1’, [10] It has long been known that, above and beyond the requirements of classic cholinergic transmission, AChE can be released in a potassium evoked, calcium-dependent manner independent of its enzymatic function and can operate as a trophic signaling molecule irrespective of any classic neurotransmission [11].

The salient part of the molecule underlying the non-enzymatic action of AChE is a 14mer peptide, ‘T14’ cleaved from the C-terminus, and leaving the parent molecule as a monomer, ‘G1’, unable to oligomerize into a characteristic tetramer, G4, due to the absence of disulphide bonds. The free T14 molecule binds at an allosteric site on the α7nAChR thereby enhancing calcium influx and triggering intracellular mobilization of the ion [12, 13]. Epidermal keratinocytes express both the α7nAChR and also selectively high levels of AChE G1 form [10]: if this profile is indeed indicative of cleaved T14 acting as an independent bioactive molecule [14] that promotes cell growth and renewal [12, 15], then perhaps this 14mer peptide could be a previously undiscovered agent promoting keratinocyte growth and proliferation.

The first aim of this study was therefore to see if T14-ir could be detected in keratinocytes using an antibody that would not recognize the parent AChE itself, and thus be readily differentiated from it. It is already known that acute light exposure induces changes AChE expression in keratinocytes [16], specifically the G1 form [5]. Hence the second aim of the study was to investigate the possibility that T14 was not only present in keratinocytes but could be regarded as an index reflecting not just age but also photo-induced aging.

## RESULTS

### Specificity of T14 antibody

**Figure 1:**
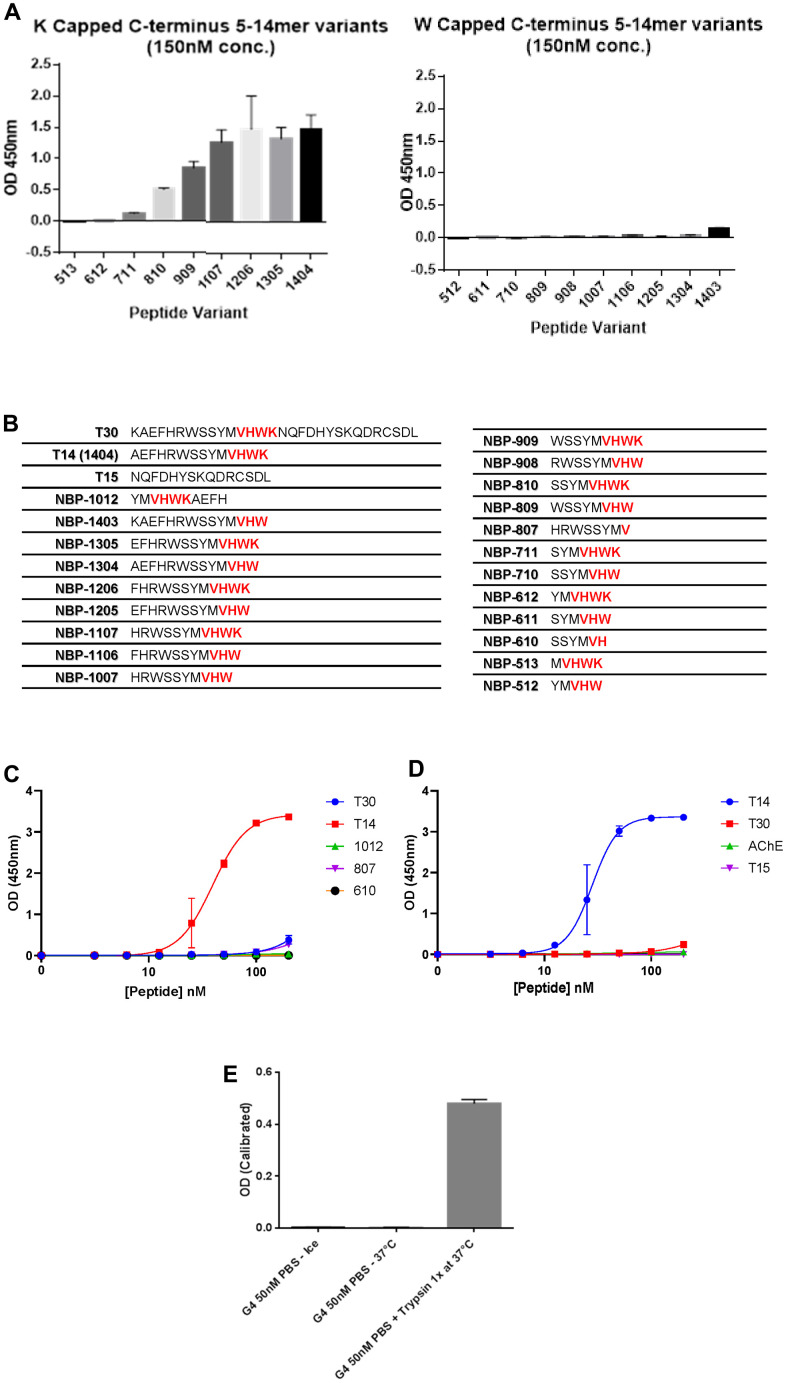
**Specificity of T14 antibody.** (**A**) Comparison of T14 antibody specificity using ELISA to detect peptides of various lengths with the C-terminus capped with W or K. (**B**) List of peptides used with amino acids sequences (epitope of the antibody -VHWK highlighted in red). (**C**) Dose-response of different peptides at the nanomolar range to determine the importance of the length and the epitope. (**D**) Dose response of T14, T30, T15 and AChE at nanomolar range. (**E**) Effect of trypsin on full AChE, cleaving T14 now detectable by the antibody.

To explore the expression of T14 in skin, a bespoke polyclonal antibody was designed against the peptide. This antibody showed a high specificity for T14 by recognizing only the epitope VHWK in the C-terminal of the peptide when the terminal lysine was exposed (Figure 1A–1C) and not within a longer sequence as in the parent AChE (Figure 1E). Moreover, there was substantially reduced immunoreactivity to a variant of synthetic T14 variants lacking the final exposed lysine residue and ending with a tryptophan (C-terminal W) (Figure 1A, 1B). An additional constraint also appeared to be the length of the N-terminal sequence: the greater the number of amino acids preceding the VHWK epitope, the more potent the antibody recognition (Figure 1A, 1B). These data indicate that the antibody binds to the terminal region of T14 and that this region is conformationally inaccessible in the larger molecules T30 and AChE. The antibody recognized neither T30, the larger peptide derived from the C-terminal of AChE, nor T15, the residual sequence following that of T14 at the C-terminus in the 30mer, (Figure 1E). AChE alone, incubated with the antibody at either 4° C or 37° C was ineffective: but when incubated with the enzyme trypsin, a signal was detected, attributable to the cleavage of T14 peptide by the protease acting at trypsin cleavage points and thus now available as an antigen to be recognized by the antibody, (Figure 1E).

### T14-ir in human skin

**Figure 2:**
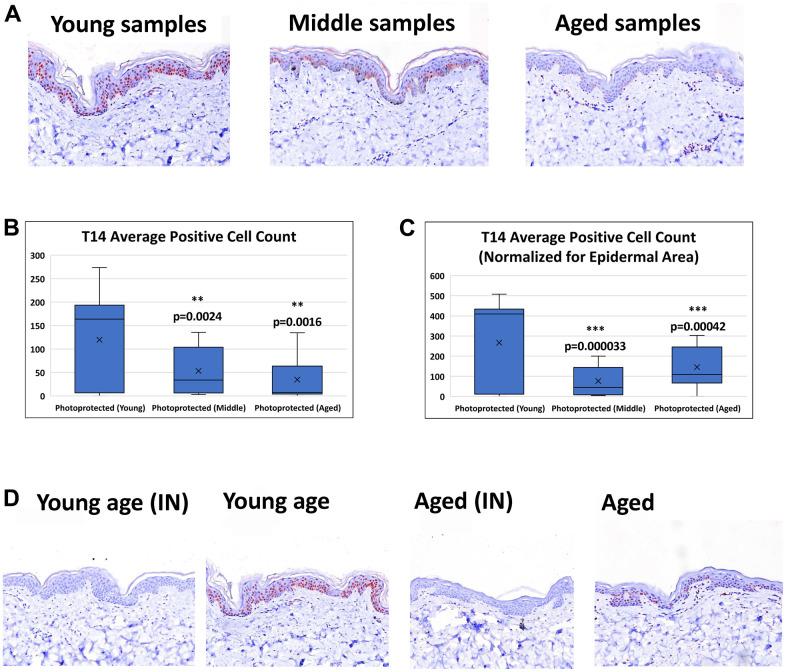
**Detection of T14 in skin.** (**A**) Immunohistochemistry of AChE T14 peptide expression is observed in both the dermis and epidermis. In the epidermis the expression is high in human skin of young subjects (16 to 31 years old), decreases in skin of middle-aged subjects (37 to 45 years old) and further declines in skin of aged subjects (50 to 70 years old). (**B**) Average number of epidermal cells with a positive AChE T14 peptide antibody stain. Young skin samples have a significantly higher expression (p value of 0.00018) of T14 positive cells compared to its expression in both middle-aged and aged skin samples. (**C**) Average number of epidermal cells with a positive AChE T14 peptide antibody stain normalized by epidermal area. Young skin samples have a significantly higher expression (p value of 0.0000015) of T14 positive cells per epidermal area compared to its expression in both middle-aged and aged skin samples. (**D**) Peptide block of T14 and anti-T14 staining of young PP skin sample and aged PP skin sample. Peptide successfully blocked T14 binding of epitope for both young and aged photo-protected skin tissues. N=10 in each group.

T14 positive cells are observed in both epidermal and dermal layers of the skin. However, this study focused on T14-epidermal expression, given its potentially critical role it the epidermal self-renewal process and skin aging pathology. The intracellular expression of T14 in the epidermal layer is seen in both the nucleus and the cytoplasm. The number of positive T14 cells is decreased in aged vs young skin (Figure 2A). Image analysis of epidermal T14 positive cells reveals a significant decrease in aged skin compared to young skin (Figure 2B). Image analysis of T14-postive cells while accounting for differences in epidermal area reinforce the observation of decreased T14 expression in aged skin (Figure 2C). Antibody specificity was further confirmed with the pretreatment of the antibody with exogenous T14 peptide successfully blocked T14 immunoreactivity in all cases (Figure 2D).

**Figure 3:**
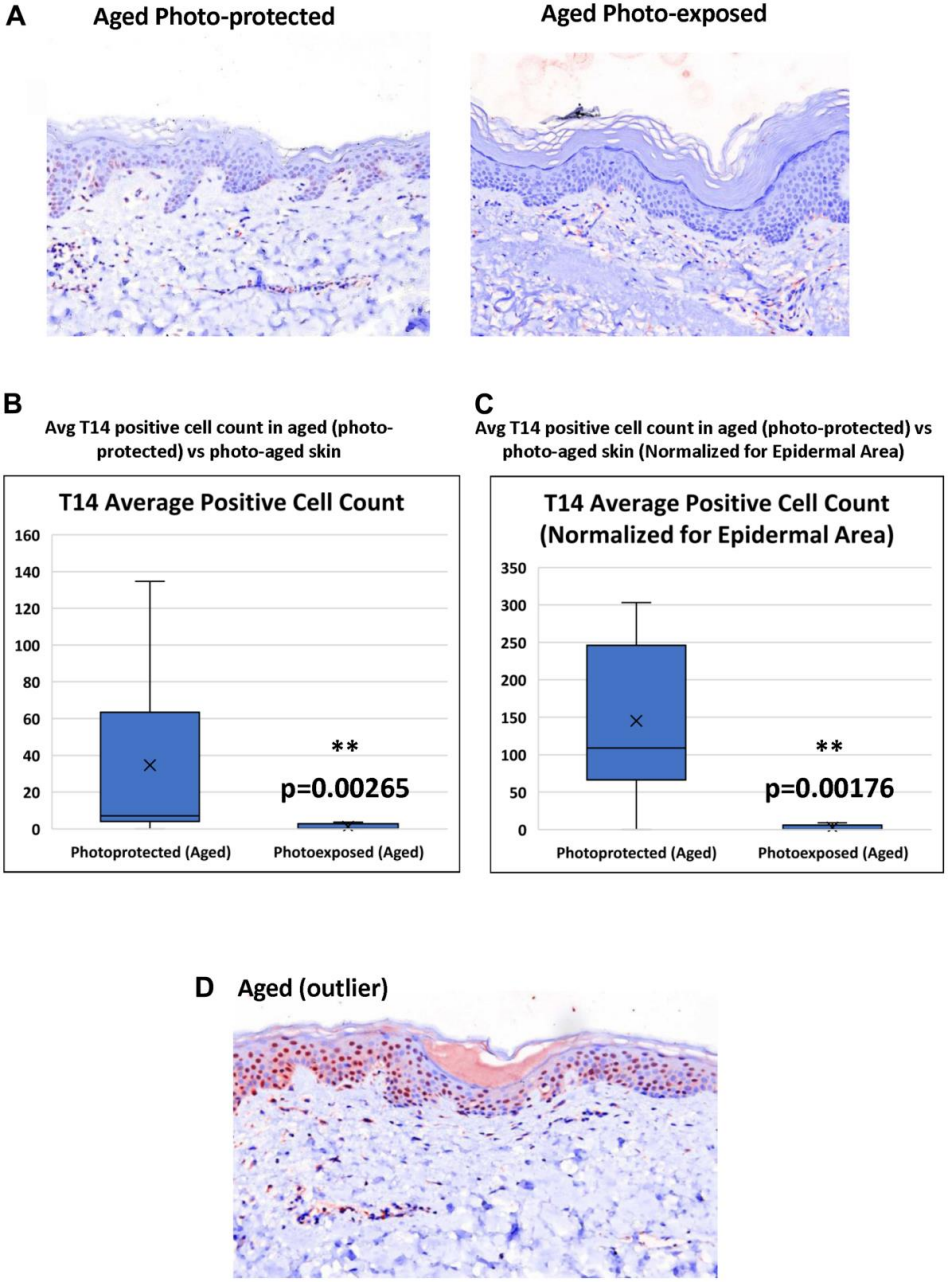
**Effect of UV-light on levels of T14 in skin.** (**A**) Immunohistochemistry of AChE T14 peptide expression is higher in aged photo-protected (50 to 70 years old) skin compared to photo-aged skin (50 to 70 years old. N=10 in each group. (**B**) Average number of epidermal cells with a positive AChE T14 peptide antibody stain. Aged (photo-protected) skin samples have a significantly higher expression (p value of 0.00265) of T14 positive cells compared to its expression in age matched, photo-aged skin samples. (**C**) Average number of epidermal cells with a positive AChE T14 peptide antibody stain normalized by epidermal area. Aged (photo-protected) skin samples have a significantly higher expression (p value of 0.00176) of T14 positive cells per epidermal area compared to its expression in age-matched photo-exposed skin. (**D**) Immunohistochemistry of AChE T14 peptide expression is higher in 1 outlier of the aged group.

T-14 expression is further decreased in age-matched photo-exposed skin vs photo-protected skin (Figure 3A). Triplicate images were collected across all samples in order to demonstrate T14 positive expression. Although, a majority of the images acquired across the photo-exposed samples demonstrated negative T14 staining, half of the panelists demonstrated some areas of minimal positivity, which gives further confidence in the reduction of T14 in photo-exposed skin. Image analysis confirmed that photo-aged skin has a significant reduction in the number of T-14 positive, epidermal cells even when epidermal area is taken into account (Figure 3B, 3C).

## DISCUSSION

The current study demonstrates the presence of the 14mer peptide T14 as an independent molecule in human skin, dermal and epidermal compartments, using immunohistochemistry with a proprietorial polyclonal antibody. The immediate issue to clarify is therefore that the signal seen is indeed attributable specifically to this peptide, and not to any wider, non-specific cross contamination, nor indeed to recognition of the parent AChE molecule itself. As shown in Figure 1, the antigens with a selective affinity for the antibody are only those sequences (i) exclusively containing the VHWK epitope where (ii) the C-terminal lysine is exposed, since capping with a tryptophan abolishes the signal, and (iii) where the sequence is at least an additional 6 residues in length at the N-terminus. As can be seen in Figure 1, responses to VHWK containing but shorter sequence peptides are weak (Harlow and Lane, Antibodies Laboratory Manual 1988). Moreover, we can discount any recognition by the antibody of related variants to T14, namely (i) the longer 30aa sequence T30 which, though containing T14 and bioactive when applied as an exogenous treatment [13], nonetheless does not allow exposure of the terminal lysine; (ii) the functionally inert 15mer at the C terminus of T30 often serving as a control peptide [13].

Further evidence that the signal seen relates to T14 only when cleaved from AChE, can be deduced by comparing the parent molecule alone, unrecognized by the antibody irrespective of temperature, with AChE incubated with trypsin: the C-terminus of AChE has identified trypsin cleavage points that would allow for the generation of T14 as an independent molecule (https://web.expasy.org/peptide_cutter/): hence incubation with this protease gives rises to cleavage products including T14 subsequently recognized by the antibody. Final confirmation for the T14 antibody specificity, and demonstrating that cross contamination with any other molecules in human skin can be discounted, is deduced from the observation that immuno-neutralization of the antibody pre-incubated with the exogenous antigen T14, abolishes the immunoreactive signal in all cases.

It has now been well established that T14 is a bioactive molecule in a range of *in vitro* and *ex vivo* preparations (Garcia-Ratés et al., 2022) with both acute [12, 15, 17, 18] and chronic [13, 17, 19] actions: its effects are due to enhancing calcium entry through binding to an allosteric site on the alpha 7 receptor, in a receptor-dependent manner [12]. This effect is particularly dominant early in life and correlates with the same timeline as enhanced, large scale neuronal activity that diminishes into brain maturity [14]. Moreover, complexing of T14 with the alpha-7 receptor, as demonstrated with Co-IP (Greenfield et al., 2022) in developing rodent brain as well as with Alpha-Lisa in post mortem human tissue (Greenfield et al., 2022), is sensitive to age and pathology and confirms that the bioactivity of T14 is via the alpha 7 receptor.

Epidermal keratinocytes have high levels of AChE G1 [10] and here we show for the first time, that the T14 profile in skin is age-dependent, declining as the rate of keratinocyte proliferation decreases, a finding consistent with a possible trophic role for T14 in the epidermis, and as reported in other preparations [12, 17].

The characteristic profile of high AChE G1 in parallel with high T14 in the developing brain has been interpreted as a failure of oligomerization into the characteristic tetrameric (G4) form, due to the absence of disulphide bonds, in turn resulting from cleavage of c-terminal peptides, including T14: G1 can thus be regarded as an indirect index of free, independently bioactive T14 [20].

In contrast to the brain, in normal circumstances in the healthy epidermis, cell growth and renewal persist into maturity, though less rapidly with older age: intrinsic and photo-aging of the human skin subsequently reflect impairment of several mechanisms, including reduced epidermal proliferation, impaired melanocyte function, and decreased collagen biosynthesis [21–23]. The aged epidermal layer is thinner due to a decrease in keratinocyte proliferation and differentiation. It is important to note that the sharpest decline in T14 expression occurred between young vs middle and young vs aged with a trending decline between middle and aged. This phenomenon has previously been also noted in a study exploring skin aging across several decades, which points to a sharper decline in genes involved in energy metabolism and epidermal cell turn over between 20’s and 30’s age groups contrasting with a clearer decline in those over 60. The higher variability seen in the middle and aged groups was noted to be due to the presence of older subjects with a “young” gene expression profile within the middle and aged groups [24].

Skin aging occurs due to an accumulation of errors at the genetic and cellular level and consequently, several proteins have been shown to change expression pattern in aged and photoaged skin: for example, collagen 17 A1 (COL17A1), a critical collagen at the dermal-epidermal junction, decreases in aged and photo-aged epidermis [25]. In addition, reduced stem cell abundance or self-renewal ability is also a feature of aged epidermis [26]. However, this list of factors is by no means exhaustive: in this regard, it is interesting to note that there was a single outlier with a very intense T14 profile in the older age group (Figure 3D). One possibility is that this particular subject’s skin aged less compared to their counterparts in this age group, ie could be classified as a “good ager” with their epidermal self-renewal process less impacted by their individual aging process. The phenomenon of “good-agers” vs “poor-agers” is well documented in cross-sectional studies investigating the role of environment, lifestyle and genetics in skin aging with monozygotic twins [27].

Nonetheless this outlier showing high T14 in the older age group prompts the questions: is the peptide a direct and inflexible marker for calendar age alone? Epidermal cell growth and proliferation is highly multifactorial and influenced by a range of environmental factors which include, for example, UV and even pollutants [10, 16, 28]. The second part of this study showed a significant difference, within the same age range, of photo-protected versus photo-exposed skin, such that T14 declined as an index not only of age, but of aging. This observation is consistent with the notion that T14 is actively involved in the actual rate of cell renew, and/or in cellular growth mechanisms, rather than merely reflecting calendar age. A role for T14 acting on the α7nAChR in the brain as a novel signaling molecule, has already been implicated in three different metastatic cancers [29] and in Alzheimer’s disease [30], where in all cases it has been posited to drive an inappropriately triggered developmental mechanism [9, 14].

Neural and epidermal tissues are both derived from the ectoderm, which suggests that the latter could well present with similar scenarios to the former, and aberrations in skin biology have already been linked to neurodegenerative disease. The skin expresses markers related to neurodegeneration such as APP1, tau PSEN1, and PARK2 [31, 32]. Moreover, a link between brain and skin has been proposed, based on markers of skin ageing [33], suggestive of some pathological skin states being indicative of greater underlying potential for CNS ageing. The neurodegenerative disorder Parkinson’s Disease has been shown to be accompanied by an increased risk of melanoma among other skin afflictions alongside the typical motor impairment [34]. Proteinopathies have also been the focus of studies such as alpha-synuclein and pTau [32]. Normal cultured human cells were found to express various isoforms of APP [35]. Excessive production of amyloid beta has been demonstrated in the skin of patients with dementia and has been shown to cause abnormalities in fibroblast biology [36]. Finally, a study by Joachim et al., (1989) showed that amyloid beta was present in skin of dementia patients more frequently than in healthy age matched controls.

The hyperproliferative keratinocyte disorder, psoriasis, could be comparable to AD, if indeed both conditions are characterized by attempts at inappropriate cell growth and renewal. Patients with psoriasis, have shown a significantly increased risk of AD when compared with healthy controls [37, 38] whilst therapeutics for psoriasis are being investigated for their possible impact on AD, with TNF blocking agents associated with lower risk of AD for [39]. Drugs already approved for use against psoriasis may be repurposed for treatment of AD [40]. Moreover, the secretory n-terminal domain of APP (sAPP) has been implicated in the regulation of keratinocyte proliferation, the underlying cause of psoriasis. Hence the current study could have eventual implications for a potential skin-based screen for AD and other degenerative diseases, based on age-matched T14 immunoreactivity.

More immediately the results suggest a possible novel approach exploring skin disorders *per se*. Eczema and psoriasis result from the excessive proliferation of keratinocytes, and since T14 might be an agent driving keratinocyte production, then local blocking with an antagonist of T14, the cyclated version NBP 14 [41–43] Garcia-Ratés and Greenfield, 2022), could offset over-production. Hence further exploration of the T14 system in the epidermis might prompt new insights into the treatment of hyperproliferative skin disorders, as well as into the mechanisms of normal skin age and ageing.

## MATERIALS AND METHODS

### Tissues

Samples consisted of formalin-fixed paraffin embedded (FFPE) skin. Skin samples included 10 skin donor samples per age group. The age groups included young (16 to 31 years old), middle-aged (37 to 45 years old), aged (50 to 70 years old) and photo-aged (50 to 70 years old). All image analysis performed for each of the age groups, including the photoaged, were from individual donors. The young, middle-aged, and aged skin were from abdominal or breast skin, while the photo-aged skin was from face, arm, and back. Skin tissues were procured from external tissue banks which included National Disease Research Interchange (Philadelphia, PA, USA) and Precision for Medicine (Bethesda, MD, USA).

### Immunohistochemistry

Immunohistochemistry was performed on young photo-protected (ages 16-31), middle photo protected (ages 37-45), aged photo protected (ages 50-70), and aged photo-exposed (ages 48-83) skin samples. All samples were paraffin embedded and sectioned to generate 5μm thick samples. Detailed procedures for standard immunohistochemical techniques were as follows. The specimens were hydrated through xylene and alcohol washes and then run through distilled water. High heat epitope retrieval was performed in a decloaking chamber, slides were cooled to room temperature, and then washed in distilled water. The subsequent steps were all performed on an IntelliPath immunostainer (Biocare Medical, Pacheco, CA, USA) including: blocking step with Peroxidazed1 Blocking reagent (Biocare Medical, cat# PX968) for ten minutes, anti-T14 primary antibody (Genosphere, Paris, France) incubation for one hour, probe and polymer incubation (Biocare Medical, cat# M4U536) for 10 minutes, Vulcan Fast Red (Biocare Medical, cat# IPK5017) chromagenic labelling of samples for fifteen minutes, and hematoxylin (Biocare Medical, cat# NM-HEM) counterstain for three minutes. Slides were then washed in water, dried in an oven at 60 degrees C for one hour, and cover slipped.

For immunoneutralization experiments, T14 antibody (1:100 dilution) was preincubated with T14 blocking peptide (10μg/mL) for one hour before application to skin samples, as described above.

### Peptides

All the peptides used in the characterisation of the T14 antibody were synthesized by Genosphere Biotechnologies (Paris, France). Acetylcholinesterase and Trypsin were provided by Sigma-Aldrich.

### T14 antibody

The antibody was synthesised by Genosphere Biotechnologies (Paris, France). Two New Zealand rabbits were used with four immunisations with keyhole limpet hemocyanin (KLH)-peptide T14-hapten: CAEFHRWSSYMVHWK; C was included to link to KHL as immunogen) over 70 days. The animals were bled four times and the bleeds pooled. The antiserum was then passed through a gravity column with covalently bound peptide support and, following washing, the antibodies were eluted in acidic buffer and the solution neutralised. Further dialysis against phosphate buffered saline (PBS) buffer and lyophilisation completed the process.

### ELISA for T14 peptide antibody

The peptides were prepared in PBS and the ELISA was performed as in Garcia-Ratés et al. 2016.

Briefly, 96-well immunoplates (NUNC) were coated with 100μl/well of sample or standard T14, covered with parafilm and incubated overnight at 4° C. The following day the sample was removed by flicking the plate over a sink with running water, and 200 μl of the blocking solution containing 5% bovine serum albumin (BSA) in Phosphate-buffered saline (PBS) was added and incubated for 2hrs at room temperature. Blocking solution was then removed and wells were washed 3 times with 300 μl of Phosphate-buffered saline with 0.1% Tween 20 (PBS-T). 100 μl of antibody, diluted 1:1000 in 1% BSA in PBS, was added and incubated for 1.5hrs at room temperature. The primary antibody was removed and wells were washed 3 times with 300 μl of PBS-T. After, 100 μl of secondary enzyme-conjugated antibody diluted 1:10 000 in 1% BSA was added and incubated for 1h at room temperature; the plate was covered with parafilm during all incubations. After 1h, the plate was washed 3 times with 300 μl of PBS-T. The addition of 3,3,5,5-tetramethylbenzidine started the colour reaction. The reaction was stopped 15 min later with stopping solution containing 2 MH2SO4, and the absorbance was measured at 450 nm in a Vmax plate reader (Molecular Devices, Wokingham, UK). For the trypsin experiment, acetylcholinesterase was pre-incubated with trypsin 1X at 37C in PBS before performing the ELISA assay.

### ELISA analysis

For the ELISA experiments, the data was represented in the figures as curves where different, known concentrations of the peptides/proteins were plotted against the respective optical density reading using an ‘exponential model’. The statistics analysis was performed with the average of the percentage values of 3 or more experiments. Comparisons between multiple treatment groups and the same control were performed by one-way analysis of variance (ANOVA) and Tukey’s post-hoc tests using GraphPAD Instat (GraphPAD software, San Diego, CA, USA). Statistical significance was taken at a p value < 0.05.

### Image analysis

Image acquisition was performed on a Zeiss microscope using a Nuance multispectral camera. Image analysis was performed using the Deconvolution Module and the FL HighPlex Module of HALO image analysis software (Indica Labs, Albuquerque, NM, USA). Image analysis was performed on all samples. To achieve quantitative results in the viable epidermis, a region of interest (ROI) was drawn along the uppermost granular layer and along the dermal epidermal junction (DEJ). Positive cell count within this area consisted of cells that demonstrated expression of T14 protein, whether in nucleus or cytoplasm. ANOVA testing was performed to determine statistical significance of changes.

### Data availability statement

No datasets were generated or analyzed during the current study.
